# Assessment of quantitative and semi-quantitative biological test methods of artesunate *in vitro*

**DOI:** 10.1051/parasite/2022019

**Published:** 2022-03-29

**Authors:** Yobouet Ines Kouakou, Roukayatou Omorou, Ibrahim Bin Said, Adeline Lavoignat, Guillaume Bonnot, Anne-Lise Bienvenu, Stéphane Picot

**Affiliations:** 1 Université de Lyon, Malaria Research Unit, ICBMS, UMR 5246 CNRS-INSA-CPE-Université Lyon1 69622 Villeurbanne France; 2 Groupement Hospitalier Nord, Institut de Parasitologie et Mycologie Médicale, Hospices Civils de Lyon 69004 Lyon France; 3 Groupement Hospitalier Nord, Service Pharmacie, Hospices Civils de Lyon 69004 Lyon France

**Keywords:** Artesunate, Malaria, Bioassay, Quantitative test, Semi-quantitative test

## Abstract

Artesunate is the current most potent antimalarial drug widely used for the treatment of malaria. Considering the emergence of artemisinin resistance, several situations may require a simple method for artesunate quantification. We thus developed a quantitative and a semi-quantitative biological method for the determination of artesunate in liquid samples. The tests are based on the measurement of samples’ antimalarial activity on *Plasmodium falciparum* 3D7 using a modified SYBR Green I drug susceptibility test. For the quantitative test, we established a standard curve that resulted from a dose–response curve and evaluated its performances using controls samples. Whereas the linear regression analysis between artesunate concentration and antimalarial activity showed promising results (linearity range 1.5–24.6 ng/mL, *r*^2^ = 0.9373), we found that artesunate content of the controls was significantly overestimated (*p* = 0.0313). For the semi-quantitative test, we compared the antimalarial activities of samples collected during permeation studies of artesunate to that of a reference (artesunate IC_50_) by statistical analysis. We demonstrated that antimalarial activities of samples from permeation tests using a powder formulation of artesunate were greater than those of samples from tests using a solution formulation. Bioassays can be simple techniques to assess artesunate in liquid samples, particularly in resource-limited settings. Comparison with reference methods is still recommended when accurate drug quantification is required.

## Abstract (fr)

L’artésunate est le médicament antipaludique le plus puissant actuellement, largement utilisé pour le traitement du paludisme. Compte tenu de l’émergence de la résistance à l’artémisinine, plusieurs situations peuvent nécessiter une méthode simple de quantification de l’artésunate. Nous avons ainsi développé un test biologique quantitatif et un test semi-quantitatif pour le dosage de l’artésunate dans des échantillons liquides. Les méthodes sont basées sur la mesure de l’activité antipaludique des échantillons sur *Plasmodium falciparum* 3D7 à l’aide d’un test de sensibilité aux médicaments SYBR Green I modifié. Pour le test quantitatif, nous avons établi une courbe standard issue d’une courbe dose-réponse et évalué ses performances à l’aide d’échantillons témoins. Alors que l’analyse de régression linéaire entre la concentration d’artésunate et l’activité antipaludique a montré des résultats prometteurs (gamme de linéarité de 1,5 à 24,6 ng/mL, *r*^2^ = 0,9373), nous avons constaté que la teneur en artésunate des témoins était significativement surestimée (*p* = 0,0313). Pour le test semi-quantitatif, nous avons comparé les activités antipaludiques d’échantillons collectés lors des études de perméation de l’artésunate à celle d’une référence (artésunate IC_50_) par analyse statistique. Nous avons démontré que les activités antipaludiques des échantillons provenant de tests de perméation utilisant une formulation en poudre d’artésunate étaient supérieures à celles des échantillons provenant de tests utilisant une formulation en solution. Les dosages biologiques peuvent être des techniques simples pour évaluer l’artésunate dans des échantillons liquides, en particulier dans les milieux à ressources limitées. La comparaison avec des méthodes de référence est toujours recommandée lorsqu’une quantification précise du médicament est requise.

## Introduction

Malaria is a catastrophe for humanity. Despite several international programs, the disease still kills more than 550,000 people each year, most of them being children from developing countries [31]. Moreover, progresses in malaria management have been stalling for the last few years. This observation is even more alarming as resistance to antimalarial drugs continues to spread. *Plasmodium falciparum* (*Pf*) resistance to artemisinin derivatives, the most potent antimalarial drugs in use, is characterized by delayed clearance of the parasite [20]. It was first described a decade ago in Western Cambodia and has since spread to South–East Asia [7, 19]. Several recent clinical studies reported the spontaneous emergence of this resistance phenomenon in Africa where its spread could lead to a public health disaster [4, 29, 31].

Artesunate, a semisynthetic derivative of artemisinin, is the first-line treatment for severe malaria when administered intravenously [31]. It can also be used orally in combination with long-lasting antimalarial drugs like piperaquine or lumefantrine for the treatment of uncomplicated malaria [31]. The widespread use of artesunate for the treatment of severe or uncomplicated malaria raises the need for therapeutic drug monitoring (TDM) [5]. Although artesunate quantification is not a priority in terms of patient management, a simple quantification method might be required for the detection of underdosed or fraudulent drugs [2, 10, 14]. It might also be useful during the early stages of developing galenic formulations, when the availability of a quantification method is necessary to study drug solubility or mucosal permeability for instance [12, 16].

Methods for artesunate quantification including UV–VIS spectrophotometric [13, 23], high performance liquid chromatography (HPLC) [22], and liquid chromatography with mass spectrometry detection (LC-MS or LC-MS/MS) [8, 26] were previously described, but they are costly and difficult to implement in malaria endemic regions. Whereas attempts were made to develop low cost methods such as lateral flow immunoassay (LFIA) [15] and enzyme-linked immunosorbent assay (ELISA) [17], they are still difficult to implement in resource-limited countries. In this context, a bioassay based on antimalarial activity measurement appears to be a suitable method for artesunate quantification in various samples. Bioassays require equipment, including incubators and plates readers, which are usually available in research and biological laboratories. They could, therefore, be used for drug quantification in laboratories not equipped with chromatographic and/or mass spectrometry systems at no additional cost. Bioassays expressed in DHA concentrations equivalents were previously described, but they were based on the microdilution radioisotope method for susceptibility test [18, 24, 25].

The purpose of our study was to develop biological methods for artesunate quantification in liquid samples using their antimalarial activity on *Pf*. The methods are based on the SYBR Green I detection technique for *in vitro* drug susceptibility assay [3]. We developed a quantitative test to perform accurate artesunate dosing in samples. We also developed a semi-quantitative test that was used to quantify artesunate in samples collected in *in vitr*o artesunate permeation studies.

## Materials and methods

### Chemicals and artesunate dilutions

Artesunate (Chemical Abstract Service CAS number: 88495-63-0; IPCA #19003AS6RII [1 kg]) was purchased from IPCA Laboratories Limited (Mumbai, India) through Hepartex (Saint-Cloud, France). All other reagents were provided by Sigma-Aldrich Merck (Saint-Louis, MO, USA) and ThermoFisher Scientific (Waltham, MA, USA). Krebs ringer buffer (KRB) was prepared by dissolving 6.8 g NaCl, 0.4 g KCl, 0.14 g NaH_2_PO_4_**·**H_2_O, 2.1 g NaHCO_3_, 3.575 g HEPES, 1.0 g D-glucose, 0.2 g MgSO_4_**ּ**·7H_2_O, and 0.26 g CaCl_2_·2H_2_O in 1 L of sterile distilled water [9]. Artesunate stock solution (1.28 mM) was prepared by dissolving drug powder into pure ethanol and stored at −20 °C.

For the quantitative test, final tested artesunate dilutions into culture medium ranged from 512 to 0.25 nM. Control samples of 15 (Q1) and 30 (Q2) nM of artesunate were prepared in culture medium and tested in triplicate during two independent *in vitro* quantitative tests.

For the semi-quantitative test, artesunate dilutions and permeation assay samples were distributed in triplicate at 25 μL per well into 96-well culture plates. The plates were left to dry overnight at room temperature in sterile conditions, sealed with adhesive plastic and stored at +4 °C until use [28]. Final tested artesunate concentrations ranged from 128 to 0.5 nM for the determination of artesunate IC_50_.

### *Plasmodium falciparum* 3D7 culture

*Plasmodium falciparum* parasites were incubated at 37 °C (5% CO_2,_ humid atmosphere) into human O^+^ erythrocytes suspended in RPMI 1640 containing phenol red, 25 mM of HEPES, 2 mM of L-glutamine, 5 g/L of Albumax^TM^, 2.1 g/L of sodium bicarbonate, 0.025 g/L of hypoxanthine, and 20 μg/mL of gentamicin (haematocrit = 5%) [27]. Parasites were synchronized immediately before use. Briefly, culture medium was discarded after centrifugation (500 ×g, 4 min). The pellet was resuspended into ten times its own volume of a 5% sorbitol solution and incubated for 10 min (37 °C, 5% CO_2_, humid atmosphere). The sorbitol solution was discarded after a washing step and the pellet resuspended in 10 mL of culture medium. Parasitemia was determined after microscopic examination of a Giemsa-stained thin blood smear.

### Quantitative test

The experimental method was based on the SYBR Green I *in vitro* drug susceptibility assay [3] and the Ring-stage survival assay (RSA) [30]. Briefly, synchronized parasites were diluted to obtain a 2% hematocrit and 0.5% parasitemia suspension [32]. The first step of the assay was to mix 700 μL of culture suspension with 100 μL of each drug dilution before incubation for 20 h (37 °C, 5% CO_2_, humid atmosphere). Thereafter, the tubes were centrifugated at 500 ×g for 3 min, 700 μL of the supernatant discarded, and the pellet washed with 1.4 mL of culture medium. The washing medium was discarded and replaced by 700 μL of fresh culture medium. The suspensions were distributed in triplicate into 96-well plates (200 μL per well) and incubated for 20 h. After incubation, plates were frozen at −20 °C for at least 24 h or at −80 °C for two hours until the SYBR Green I assay was performed.

### Permeation experiment and semi-quantitative test

Samples from a permeation test conducted during the simultaneous course of an independent study were evaluated using the semi-quantitative test. The permeation test was designed to evaluate artesunate permeability on a human mucosal cell culture. Artesunate was formulated as a solution (0.75 μg/mL) and as a powder mixture (20 μg/mg, artesunate/corn starch). Aliquots of the solution (0.5 mL, equivalent to 0.375 μg of artesunate) and powder formulation (10 mg, equivalent to 200 μg of artesunate) were added to the donor chamber and 1.5 mL of KRB (37 °C) to the receptor chamber. The plates were incubated for 4 h under orbital shaking (37 °C, 5% CO_2_, humid atmosphere, 100 rpm). Samples (200 μL) were collected from the receptor chamber at fixed time intervals and immediately replaced with fresh KRB (37 °C). Samples were frozen at −80 °C until semi-quantitative tests.

The effect of KRB on *Pf* parasites was investigated. KRB dilutions in culture medium were mixed with *Pf* parasites (0.5% parasitemia, 2% hematocrit, unsynchronized), incubated for 40 h (37 °C, 5% CO_2_, humid atmosphere), then frozen at −80 °C for at least 2 h before the SYBR Green I assay. The final tested KRB concentrations were 0, 12.5, 25, 50 and 100% (v/v) leading to a significant increase in osmolarity of medium from 308 to 631 mOsm/L.

For the semi-quantitative test, unsynchronized *Pf* culture was diluted to obtain parasitemia of 0.5% and a hematocrit of 2%. An amount of 200 μL of this suspension was distributed per well into microplates predosed with artesunate dilutions (128–0.5 nM) and samples collected during the permeation tests. The microplates were incubated for 40 h (37 °C, 5% CO_2_, humid atmosphere) and then frozen at −20 °C for at least 24 h or −80 °C for 2 h until the SYBR Green I assay was performed.

### Statistical analysis

All data management was performed using GraphPad Prism version 9.2.0 and 9.3.1 software [33]. Statistical analyses including the Mann–Whitney test, one sample Wilcoxon test, nonlinear regression (four parameters, variable slope), simple linear regression, and regression slope test were performed with a significance threshold of 0.05.

For the quantitative test, a parasite growth inhibition curve was obtained using nonlinear regression analysis. The linear portion of the curve was visually located by plotting the parasites growth (Fluorescence Unit = FU) against the Log_10_ artesunate concentration and analyzed by simple linear regression. A regression slope test was performed to conclude on the linear relationship between artesunate concentration and parasite growth. The artesunate concentration of a control sample (Q) was calculated using the equation of the linear regression analysis.

For the semi-quantitative test, artesunate half maximum inhibitory concentrations (IC_50_) were calculated by non-linear regression analysis and presented as the mean ± standard deviation (SD). The artesunate semi-quantitative test was performed by statistical comparison of the antimalarial activities of samples from the permeation tests and the antimalarial activity of artesunate at approximately its IC_50_.

## Results

### Quantitative test

**Figure 1:**
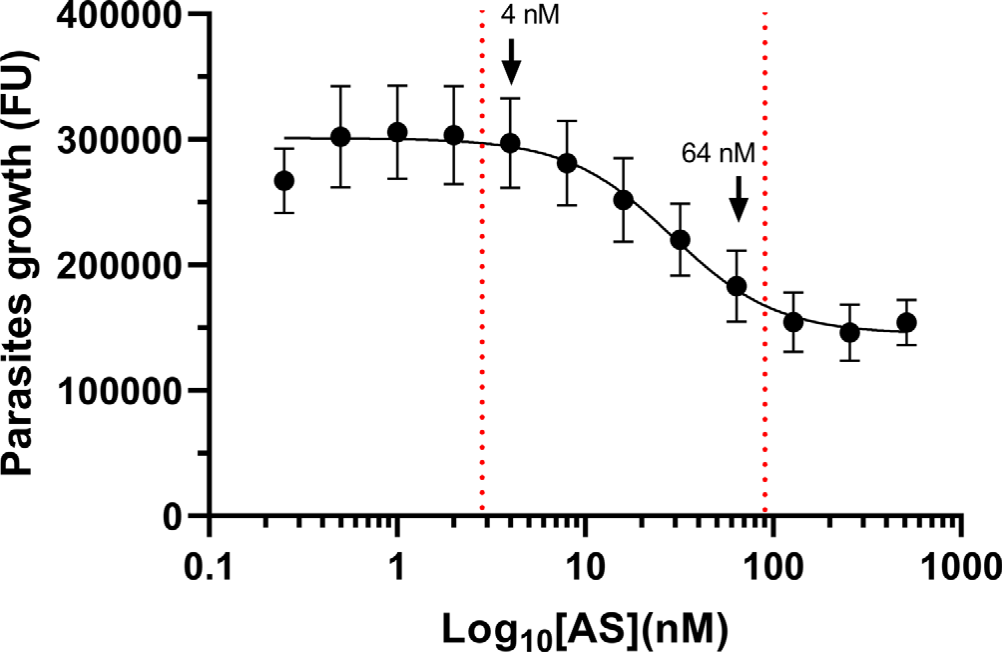
*Parasite growth inhibition curve with artesunate concentration for the quantitative test.* This curve resulted from the analysis of data sets from nine independent assays using a four-parameter nonlinear regression. Each concentration was tested in triplicate in each independent assay. The *X*-axis was transformed into logarithm to the base 10 to better visualize the linear portion of the curve. Data are presented as the mean ± SD (*n* ≥ 9). FU = fluorescence unit; Log_10_ = logarithm to the base 10; [AS] = artesunate concentration; nM = nanomolar; Red-dotted lines = indicate linearity interval.

**Figure 2:**
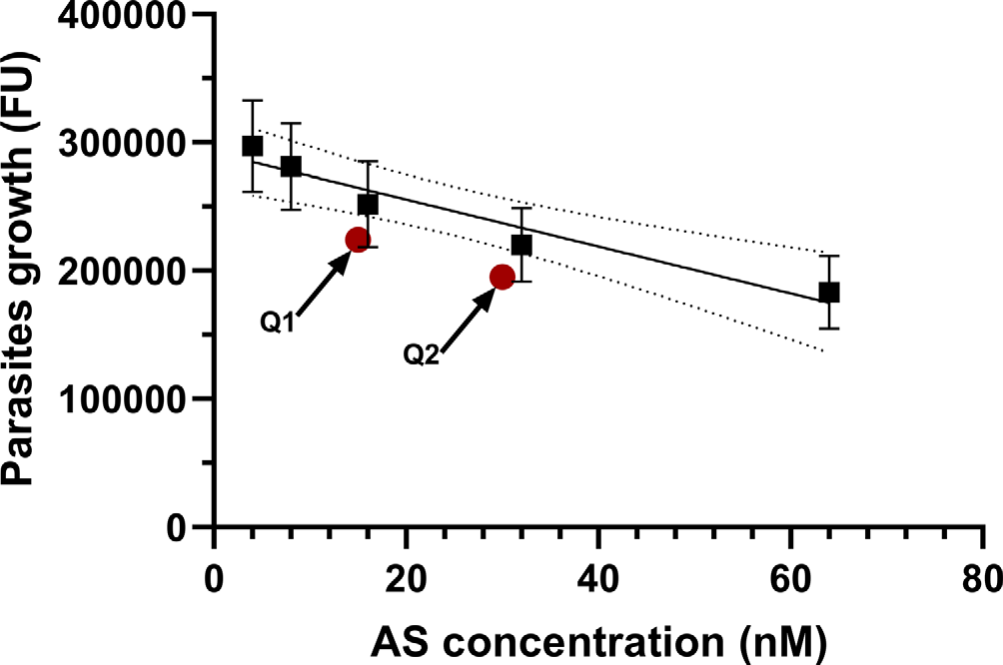
*Standard curve of the quantitative test with the 95% confidence interval for artesunate controls samples*. The equation of the curve (■) was *y* = −1831*x* + 292150 (*r*^2^ = 0.9373). The antimalarial activities of the controls (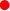), plotted against their respective expected concentrations (Q1 = 15 nM and Q2 = 30 nM), fell outside the 95% confidence interval of the curve. Data are presented as mean ± SD (*n* ≥ 6). FU = fluorescence unit; AS = artesunate; nM = nanomolar.

Quantitative tests performed in triplicate for each concentration were repeated nine times. A four-parameter nonlinear regression analysis was used to synthetize the data set and obtain a parasite growth inhibition curve with increasing artesunate concentrations (Fig. 1). A linear relationship between parasite growth inhibition and artesunate concentration was demonstrated for concentrations ranging from 4 to 64 nM (1.5–24.6 ng/mL). The equation of the linear curve was *y* = −1831*x* + 292150, its *r*^2^ = 0.9373 and its slope significantly different from zero (*p*-value = 0.0068). Artesunate concentrations calculated according to the linear curve were 37.15 ± 1.35 nM for Q1 (expected concentration = 15 nM) and 53.07 ± 2.42 nM for Q2 (expected concentration = 30 nM). The median experimental concentrations of the controls were significantly different from their theoretical values (*p*-values = 0.0313) (Table 1). Plotting the antimalarial activities of the controls against their expected concentrations (15 and 30 nM) showed that they fell outside the 95% confidence interval of the standard curve (Fig. 2).

**Table 1:** Validation data of the standard curve using controls samples of fixed artesunate concentrations.

Expected concentrations (nM)^a^	Experimental concentrations^b^ (nM)	CV^c^ (%)	Error (%)	*p*-Value^d^
15.00	37.15 ± 1.35	3.63	147.68	0.0313
30.00	53.07 ± 2.42	4.57	76.89	0.0313

a nM, nanomolar.

b Data are expressed as mean ± SD (*n* = 6).

c CV, coefficient of variation.

d The median experimental concentrations of the controls were significantly different from their theoretical respective values by a one sample Wilcoxon test.

### Semi-quantitative test

**Figure 3:**
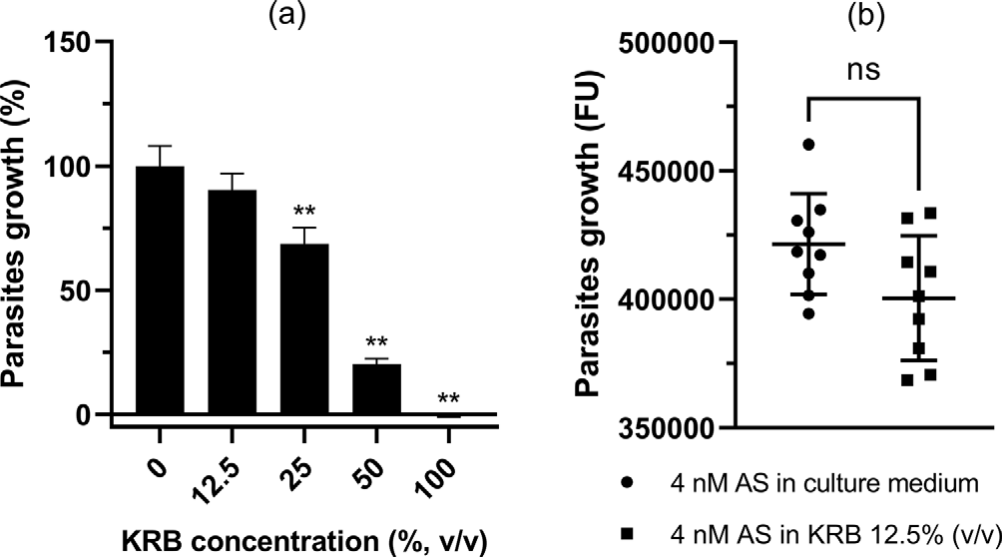
*(a) Impact of KRB on Pf growth and (b) antimalarial activity of artesunate at 4 nM prepared in culture medium or 12.5% KRB in culture medium for the semi-quantitative test.* KRB concentrations above 12.5% (v/v) significantly reduced parasites growth. All samples containing KRB were therefore diluted into culture medium before use to limit its impact. Each concentration was tested in triplicate in two independent assays. Data are presented as the mean ± SD (*n* = 6). There was no statistical difference between the antimalarial activities of 4 nM artesunate solutions prepared in culture medium and 12.5% (v/v) KRB. Each sample was tested in triplicate in three independent assays. Data are presented as mean ± SD (*n* = 9). KRB = Krebs ringer buffer; AS = artesunate; FU = fluorescence unit; ns: non-significant. Statistical analyses were performed with GraphPad Prism software, Mann–Whitney test, *α* = 5%, *p*-value (**) = 0.0022.

Semi-quantitative preliminary tests were repeated 10 times for the determination of artesunate IC_50_ within testing conditions and two times for the determination of KRB effect on *Pf* parasites. Using nonlinear regression analysis of the data set of each individual assay, artesunate IC_50_ was determined at 4.5 ± 0.9 nM. We demonstrated that KRB significantly reduced *Pf* growth for concentrations above 12.5% (v/v) which was the highest concentration providing non-toxic osmolarity (348 mOsm/L) (Fig. 3a). Importantly, antimalarial activity of artesunate at 4 nM in KRB (12.5%, v/v) was not statistically different from artesunate at 4 nM in culture medium (*p*-value = 0.0939) (Fig. 3b). Artesunate at 4 nM (IC_50_) in culture medium was therefore used as the reference condition to evaluate antimalarial activity of samples from the permeation tests. To limit the impact of KRB, all samples were diluted in culture medium to final KRB concentration of 12.5% (v/v) before performing the artesunate semi-quantitative test.

**Figure 4:**
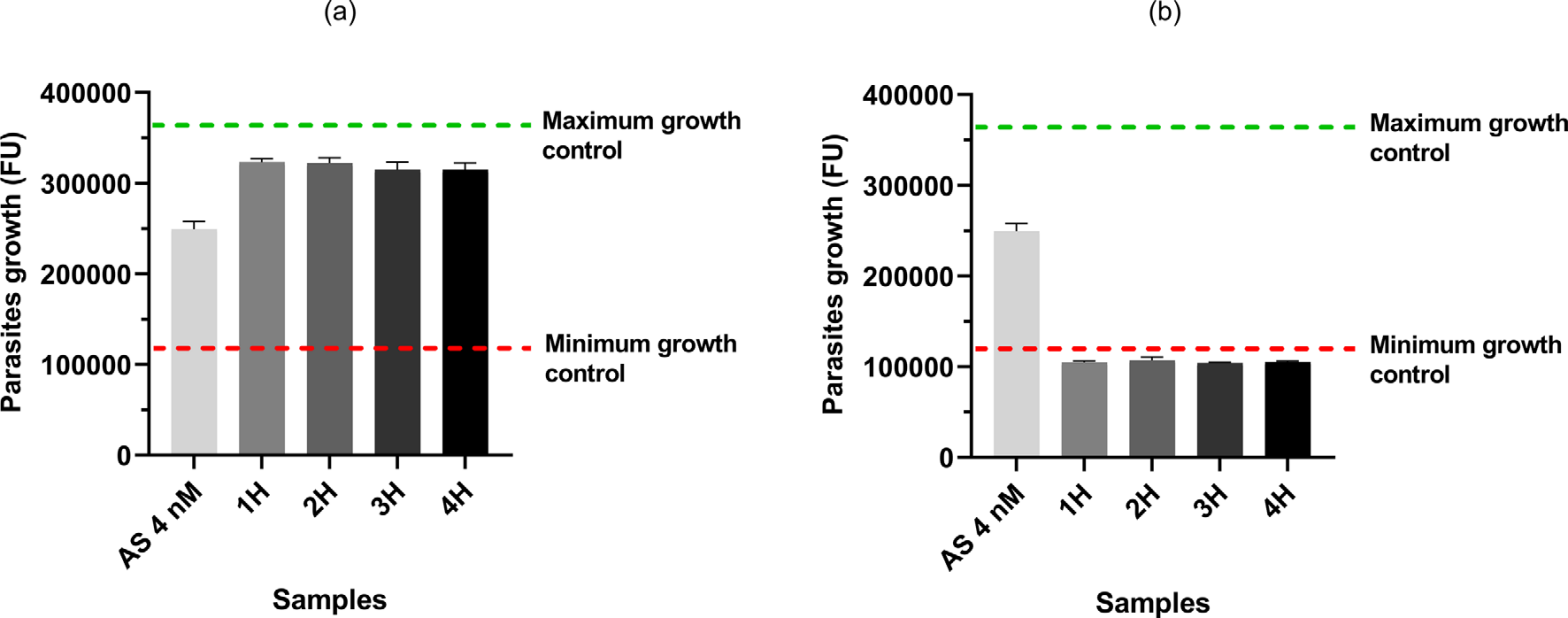
*Antimalarial activity of the reference condition (AS 4 nM) and samples from (a) the permeation test using artesunate solution and (b) the permeation test using artesunate powder.* Each sample was tested in triplicate. Data are presented as mean ± SD (*n* = 3). AS = artesunate; FU = fluorescence unit; Green dotted-line = threshold value for maximum parasite growth (fluorescence of parasites incubated without artesunate and KRB during the semi-quantitative test); Red dotted-line = threshold value for minimum parasite growth (parasite fluorescence at the beginning of the test).

Antimalarial activities of samples from two different permeation tests were evaluated using the semi-quantitative test. For the first permeation test using artesunate formulated as a solution (SOL), the antimalarial activity of the reference condition on *Pf* was significantly higher than the activities of the samples collected at hour 1 to hour 4 (H1 to H4, *p*-values = 0.0091) (Fig. 4a). The drug concentrations in these samples were therefore lower than artesunate IC_50_ (4 nM). For the second permeation test using artesunate as a powdered formulation (POWDER), the antimalarial activity of the reference condition was significantly lower than the activities of the samples collected at H1 to H4 (*p*-values = 0.0091) (Fig. 4b). Moreover, the fluorescence signals of the POWDER samples were similar to that of the minimum growth control (MGC, parasites DNA signal before incubation). The drug concentrations in these samples were therefore higher than artesunate IC_50_ and induced a total inhibition of parasites growth during the test.

## Discussion

Developing a method for artesunate quantification has multiple shortcomings because the drug is poorly soluble and readily hydrolyses in dihydroartemisinin (DHA) in aqueous acidic and neutral media [1, 11]. Several previous attempts have been made to develop simple quantitative methods for artesunate dosage including immunoassay-based tests (LFIA, ELISA) [15, 17, 21]. While some of them showed good performances, they are still difficult to implement in resource-limited countries. In this context, we aimed to develop a simple quantitative method for artesunate determination in samples using their antimalarial activities. Based on previously published data, we modified the SYBR Green I drug susceptibility assay to develop the quantitative test [6]. The RSA-based test conditions, namely the synchronization of parasites and the washing step at mid incubation time, allowed the flattening of the sigmoidal curve and a wider linearity interval. We evaluated the performances of the method by using controls samples of artesunate. Despite the promising results of the linear regression analysis, we had an overestimation of artesunate content in the controls probably related to the variation in *Pf* growth rate during the test: concentrations of the controls, calculated using the linear curve, were indeed estimated to be significantly greater than their expected values. To limit *Pf* growth rate variability and optimize the performances of this quantitative bioassay, we recommend to test in a single assay the standard curve and the samples. A similar bioassay using a modification of the microdilution radioisotope technique was previously developed [25]. It showed good performances considering the standard curves spanning over a large concentration interval (2.5–100 ng/mL, *r*^2^ > 0.98) and the strong correlation with HPLC. However, the method was only dedicated to serum and plasma, and results were expressed as DHA concentration equivalents.

In this study, we also developed a semi-quantitative method to analyze artesunate samples. It aimed to compare the antimalarial activity of unknown samples to a reference which concentration approximated artesunate IC_50_. The tested samples were collected during *in vitro* permeation assays measuring the diffusion of the drug on a human mucosal cell culture, from the donor to the receptor compartment filled with KRB [9]. These experiments were part of a proof-of-concept study to assess the mucosa as an alternative dosing site for malaria pre-referral treatment in young children. All permeation samples were diluted in culture medium prior to the semi-quantitative test as we found KRB to reduce parasites growth for concentrations above 12.5% (v/v). This reduction of *Pf* growth was likely linked to the hyperosmolarity of these KRB dilutions. Results for the semi-quantitative tests were obtained using a 4 nM artesunate solution in culture medium as reference. For the permeation test using artesunate formulated as a solution, the antimalarial activities of the samples were statistically lower than the reference antimalarial activity, whereas the antimalarial activities of the permeation samples using artesunate as a powder were statistically higher than the reference. We then concluded that artesunate as a powder had a better diffusion capacity across a semipermeable membrane compared to artesunate as a solution. This semi-quantitative test was demonstrated to be simple and useful for the comparison of antimalarial activities of various samples. We also identified the matrix of samples to be an important parameter in the test development. The evaluation of the matrix impact on the semi-quantitative test is therefore an essential prerequisite before implementing the test.

To conclude, the biological tests based on the measurement of antimalarial activity may be useful for evaluation of artesunate in various liquids, but comparison with reference methods including liquid chromatography with mass spectrometry detection is still recommended, especially if the assay is used for clinical purposes.

## Conflict of interest

The authors have no competing interest to declare.
